# Preliminary investigation of family caregiver burden and oral care provided to homebound older patients

**DOI:** 10.1002/cre2.415

**Published:** 2021-03-08

**Authors:** Yuki Ohara, Masanori Iwasaki, Keiko Motokawa, Hirohiko Hirano

**Affiliations:** ^1^ Research Team for Promoting Independence and Mental Health Tokyo Metropolitan Institute of Gerontology Tokyo Japan; ^2^ Dentistry and Oral Surgery Tokyo Metropolitan Geriatric Hospital Tokyo Japan

**Keywords:** caregiver, home care service, homebound, oral hygiene

## Abstract

**Objectives:**

Family caregivers play an important role in maintaining the oral health of homebound older adults. Thus, this preliminary study investigated family caregivers' burdens and the oral care they provide to homebound older patients.

**Material and Methods:**

A cross‐sectional survey was conducted. A questionnaire was distributed to 230 family caregivers of homebound older patients. We used the Japanese version of the Zarit Burden Interview (J‐ZBI) to measure caregiver burden. The cut‐off score for the J‐ZBI was 21 points. Caregivers with a care burden score below 21 points formed the mild group, while those scoring 21 points or more were included in the moderate/severe group. The differences between the groups were examined. The implementation status of oral care was assessed by the amount of time caregivers spent providing oral care and related concerns. The degree of independence for homebound older patients was measured using the Barthel Index. Multiple logistic regression analyses were conducted to determine the factors associated with the severity of caregiver burden.

**Results:**

A total of 114 caregivers returned the questionnaires by mail (response rate: 49.6%). The moderate/severe care burden group represented 80.7% of the caregivers. A multiple logistic regression analysis revealed that the level of patient independence and time spent performing oral care were significantly associated with the severity of caregiver burden.

**Conclusions:**

The results show that family caregivers experiencing high caregiver burden spent less time providing oral care than caregivers who reported less caregiver burden. Thus, support for oral health management provided by oral health professionals is considered necessary for family with a high caregiver burden of homebound older patients.

## 1 INTRODUCTION

Oral health is determined by multiple factors within older populations (Wong et al., 2019). Comprehensive oral health management is necessary for homebound older patients who require long‐term care in their daily lives, as previous studies have reported that they have dental care needs such as periodontal treatment and denture replacement (Gluzman et al., 2013; Ornstein et al., 2015). Furthermore, when older people become care‐dependent, their oral hygiene status usually worsens and receives less attention (Hoeksema et al., 2017; Wong et al., 2019).

Appropriate oral hygiene reduces the frequency of pneumonia and fever among patients who require long‐term care (Yoneyama et al., 2002). Since aspiration pneumonia is one of the most common causes of death in bedridden older patients (Kaplan et al., 2002), the importance of older adults receiving oral care from dental professionals has been recognized (Adachi et al., 2007; Barnes, 2014). However, reports on professional dental interventions for older adults have focused on how nursing home residents are impacted, and findings regarding their effects on homebound older patients are scarce. The major issues that affect older adults receiving medical care at home include infection, malnutrition, and cognitive impairment, which impose heavy burdens on the patients, their relatives, and medical professionals (Locher et al., 2008; Ornstein et al., 2015; Yokobayashi et al., 2014). However, healthcare providers, including dental hygienists, are not always available to visit patients' homes. Therefore, homebound patients may have less access to medical care than patients in nursing homes. The long‐term care insurance system in Japan allows a dental hygienist to visit the home of a homebound patient to provide oral health management services up to four times a month (Zaitsu et al., 2018), which is an insufficient number of visits to provide patients with complete oral health management. Therefore, family caregivers provide much of the support for daily oral health.

Caregiver burden has become an evident problem among family caregivers (Bekdemir & Ilhan, 2019). Family caregivers are defined as relatives who provide care free‐of‐charge to individuals with chronic or debilitating conditions (Bekdemir & Ilhan, 2019). Family members play important roles in caring for the sick and those unable to take care of their own needs (Bekdemir & Ilhan, 2019). It is presumed that oral care is likely to cause caregiver burden because basic oral care is an essential part of daily life. Therefore, this preliminary study aimed to determine the association between caregiver burden and the implementation status of oral care among homebound patients.

## 2 MATERIALS AND METHODS

### 2.1 Study design and participants

A cross‐sectional survey was conducted between December 2019 and March 2020, using an anonymous self‐administered questionnaire, with the cooperation of the organizations that agreed with the purpose of this research. The questionnaires were sent to an incorporated nonprofit organization that provides support for family caregivers and dentists and dental hygienists who provide home‐visit dentistry in Tokyo and its surrounding areas. Next, the questionnaires were distributed directly to 230 family caregivers of homebound older patients. The completed questionnaires were returned to the authors by mail. Family caregivers were informed of the aims of the investigation and their consent was obtained. This study was approved by the ethics committees of the Tokyo Metropolitan Institute of Gerontology (approval No. R1‐39).

### 2.2 Measures

We used the Japanese version of the Zarit Burden Interview (J‐ZBI) to measure caregiver burden (Arai et al., 1997; Zarit et al., 1980). The J‐ZBI is a 22‐item self‐administered questionnaire that measures subjective caregiver burden and has demonstrated high validity; higher scores indicate a greater care burden (Arai et al., 1997). In the present study, the cut‐off score used for the J‐ZBI was 22 points according to the previous study (Srivastava et al., 2016). Caregivers with a care burden score below 22 points were included in the mild group, and caregivers scoring 22 points or more were included in the moderate/severe group. Demographic information was collected from the caregivers and included their ages, relationship to the patient, years of caregiving, the patients' ages, and basic activities of daily living assessed using the Barthel Index (BI) (Mahoney & Barthel, 1965). Higher BI scores indicated higher levels of patient independence. Information was also collected regarding the amount of time caregivers spent performing oral care per occurrence, interest in performing oral care, and concerns about providing oral care (see Data S1).

### 2.3 Statistical analyses

Statistical analyses were performed using SPSS 25 (IBM Corp., Armonk, NY). The level of significance was set at *α* = 0.05. First, we compared the demographic characteristics and status of oral care between the mild and moderate/severe care burden groups using a *χ* ^2^ test and Mann–Whitney *U* test. A logistic regression analysis was performed to examine the factors associated with caregiver burden. The results of the estimations are shown as odds ratios (ORs) with 95% confidence intervals (CIs).

## 3 RESULTS

**FIGURE 1:**
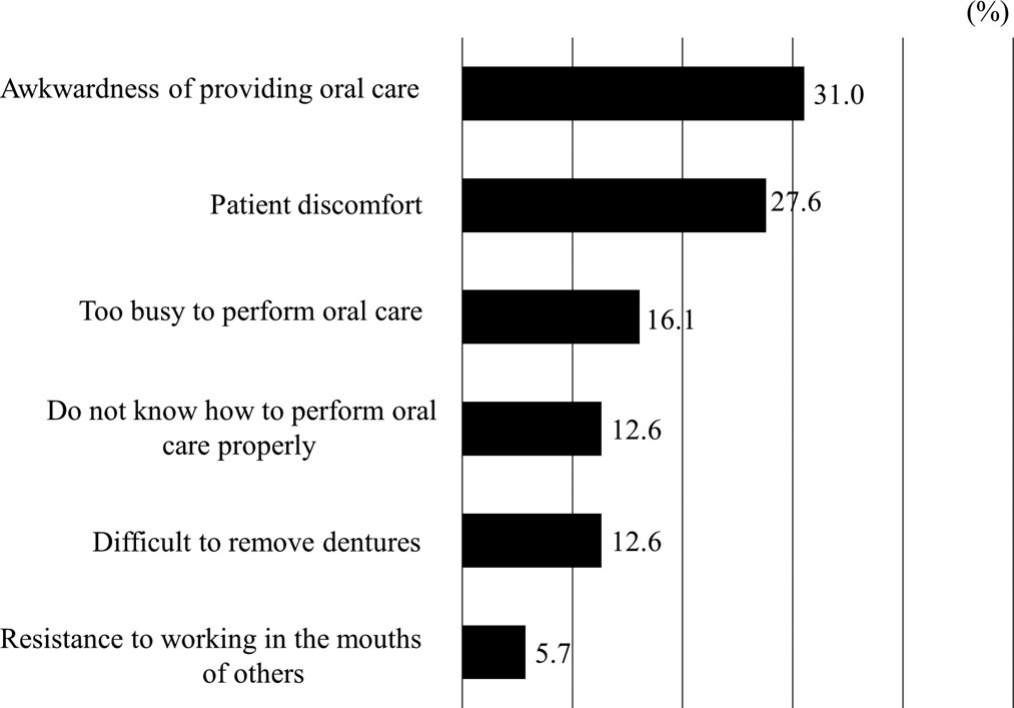
Caregivers' concerns during oral care (multiple answers)

Of the 230 questionnaires distributed, 114 were returned (response rate: 49.6%). The characteristics of the caregivers and patients are shown in Table 1. The median ages of all caregivers and homebound patients were 70 and 84 years, respectively. Regarding gender, 34 of the caregivers were male and 80 were female. As for the caregivers' relationships to the patients, 49.1% were spouses and 43.9% were their adult children. The moderate/severe care burden group comprised 80.7% of the caregivers. Figure 1 shows the results of responses to caregivers' concerns during oral care, in which caregivers could select multiple answers. The most frequent responses were “awkwardness of providing oral care” (31.0%), followed by “patient discomfort” (27.6%), “too busy to perform oral care” (16.1%), and “do not know how to perform oral care properly” (12.6%).

**TABLE 1:** Characteristics of caregivers and patients according to caregiver burden

			Caregiver burden	
	Total *N* = 114	Mild *n* = 22	Moderate/Severe *n* = 92	*p*‐value^a^
Caregivers				
Age, years	70 (62–76)	69 (62–75)	70 (62.5–75.6)	0.585^b^
Gender, female	80 (70.2)	13 (59.1)	67 (72.8)	0.206^c^
Relationship with the patients				
Spouse	56 (49.1)	12 (54.5)	44 (47.8)	0.351^c^
Children	50 (43.9)	10 (45.5)	40 (43.5)	
Others	8 (7.0)	0 (0)	8 (8.7)	
Duration of caregiving, months	73 (36–120)	90 (36–120)	73 (36–120)	0.835^b^
Interest in oral care for patient	94 (92.2)	22 (100)	72 (90.0)	0.196^d^
Time spent performing oral care, minutes per time	5 (0–10)	5 (5–15)	2 (0–10)	0.021^b^
Patients				
Age, years	84 (74–89)	81 (75–87.5)	84 (74–89)	0.668^b^
BI total score, points	35 (5–70)	5 (0–15)	45 (15–75)	<0.001^b^

*Note*: Values are median (interquartile range) for continuous variables or *n* (%) for categorical variables.

Abbreviations: BI, Barthel index; J‐ZBI, the Japanese version of the Zarit Burden Interview.

^a^ *p*‐value for the comparison between groups.

^b^ Mann–Whitney *U* test.

^c^ Chi‐square test.

^d^ Fisher's exact test.

Comparisons between the two caregiver burden groups are shown in Table 1. In the moderate/severe group, patients had significantly higher BI scores and caregivers performed oral care for shorter periods compared to the mild group. According to a multiple logistic regression analysis, the BI score (OR = 1.036, CI: 1.010–1.062) and time to perform oral care (OR = 0.919, CIs: 0.844–1.000) were significantly associated with the severity of caregiver burden (Table 2).

**TABLE 2:** Multivariate model for the association between the mild and moderate/severe groups of caregiver burden

	OR	95% CI	*p*‐value
BI total score (per one increase)	1.036	1.010–1.062	0.006
Time spent performing oral care (minutes; per one increase)	0.919	0.844–1.000	0.049
Age of caregiver (per one increase)	1.009	0.956–1.065	0.750
Sex of caregiver (0: male. 1: female)	1.206	0.345–4.220	0.769

Abbreviations: BI, Barthel index; CI, confidence interval; OR, odds ratio.

## 4 DISCUSSION

To the best of our knowledge, this preliminary study is the first to compare the implementation of oral care by caregiver burden among family caregivers of homebound older patients, even though it was conducted via a questionnaire survey that made it easy to collect data for a relatively small sample size.

In this study, approximately 80% of family caregivers who rated their caregiver burden as moderate or severe spent significantly less time providing oral care and cared for patients with higher levels of independence than caregivers who perceived their caregiver burden as mild. Generally, family caregivers of older adults with low BI scores may experience greater caregiver burden because lower BI scores indicate that older patients require nursing care and additional long‐term support in their lives (Mahoney & Barthel, 1965). However, in the present study, caregivers of older adults with a relatively high level of independence reported a higher level of caregiver burden. This is possibly because it influenced the long‐term care insurance system in Japan, which expands the range of services available as the to the severer the degree of nursing care (Tamiya et al., 2011). For example, family caregiver burden was relatively low since the nursing care service sufficiently by the public assurance system could be provided if the independence of homebound older patients was low. On the other hand, family caregivers for homebound older patients with relatively high levels of independence showed that they may not have time to provide oral care due to greater time constraints and physical burden of caregiving. It is necessary to investigate the relationship between the basic activities of living of homebound older patients and family caregiver burden since reports from previous studies are still insufficient.

In the present study, most respondents indicated they were interested in oral care. Therefore, we considered this as evidence that family caregivers were aware of the importance of oral care. However, caregivers expressed oral care concerns, such as homebound patients' refusal, how to provide oral care, time constraints, and difficulty. A qualitative survey of nursing home personnel in Australia reported a lack of time for oral care as a barrier to its delivery. The findings from that study were consistent with our results, although the caregivers and methodologies of the studies differed (Patterson Norrie et al., 2020). Homebound older patients usually have poor oral hygiene, and lack of access to dental care is one of the biggest issues regarding health problems (Gluzman et al., 2013; Ornstein et al., 2015; Wong et al., 2019). Older adults requiring long‐term care have a high need for oral health maintenance, and moreover, most of their family caregivers want home‐based dental care (Ornstein et al., 2015). Thus, oral health management by oral health professionals to complement oral care by family caregivers is considered necessary. Previous studies have also shown the effectiveness of educational interventions on oral hygiene to institutional personnel, and appropriate interventions by dental professionals to address the unmet oral health needs of homebound patients are needed as well (Khanagar et al., 2015; Zenthöfer et al., 2016). Furthermore, as homebound older patients have other health issues, such as comorbidities and the need for medications, collaboration with multioccupational groups is ideal for proper dental care provision (Ornstein et al., 2015; Wong et al., 2019).

The effectiveness of oral care in preventing aspiration pneumonia has been confirmed, but the problem of undernutrition has been indicated as a risk of pneumonia, especially in bedridden elderly people receiving oral care (Matsusaka et al., 2018). It has also been reported that homebound older patients have problems with nutritional status and dietary habits, and family caregivers play a crucial role in their improvement. Since the oral cavity is the first organ for nutrient intake, a comprehensive team approach that addresses both the nutrition and oral health needs of homebound older adults may be warranted (Hestevik et al., 2020; Hirakawa et al., 2013). Previous reports regarding the nutritional issues encountered by homebound older patients were limited qualitative studies, and the evidence regarding oral care was inadequate. Further studies are needed to validate the effects of appropriate oral and nutritional interventions on homebound older patients.

One strength of this study is that it highlights the association between the provision of oral care and caregiver burden for family caregivers of homebound older patients in Japan and provides novel findings. Promoting the oral health of homebound older adults with inconsistent access to dental services is a challenge that needs to be addressed worldwide as the population ages.

The study's limitations include its small sample size and the measures that were used due to the preliminary nature of the study. However, poor oral health associated with aging is a worldwide concern, and the findings from the present study that examined the status of homebound older patients and their caregivers in Japan can provide useful information for countries that will have aging populations in the future. Second, we cannot rule out the possibility of selection bias because the questionnaire used in this study was distributed via cooperating agencies, and our findings were derived from a survey with an approximate response rate of only 50%. For example, caregivers with severe care burden or who were not interested in oral care were less likely to respond to the survey. Thus, the current results may underestimate the association between oral care and caregiver burden. Third, caregiver burden has also been reported to impact family caregivers' mental health (Srivastava et al., 2016). Fourth, the oral health status of homebound older adults was not evaluated because the study relied solely on a questionnaire. In the future, it will be necessary to conduct a study that includes patients' oral health status with a larger sample size.

In conclusion, the present results show that family caregivers with a high caregiver burden spent less time providing oral care than caregivers who reported less caregiver burden.

## CONFLICT OF INTEREST

The authors declare no conflicts of interest.

## AUTHOR CONTRIBUTIONS

Yuki Ohara conceived the study, curated the data, and wrote the original draft of the manuscript. Yuki Ohara and Masanori Iwasaki did the formal analysis and visualized the data. Yuki Ohara, Masanori Iwasaki, Keiko Motokawa, and Hirohiko Hirano validated the data and reviewed and edited the manuscript.

## Supporting information


**Data S1**. Contents of the questionnaire.

## Data Availability

The data that support the findings of this study are available upon request from the corresponding author. The data are not publicly available due to privacy or ethical restrictions.

## References

[cre2415-bib-0001] Adachi, M. , Ishihara, K. , Abe, S. , & Okuda, K. (2007). Professional oral health care by dental hygienists reduced respiratory infections in elderly persons requiring nursing care. International Journal of Dental Hygiene, 5(2), 69–74. 10.1111/j.1601-5037.2007.00233.x 17461957

[cre2415-bib-0002] Arai, Y. , Kudo, K. , Hosokawa, T. , Washio, M. , Miura, H. , & Hisamichi, S. (1997). Reliability and validity of the Japanese version of the Zarit caregiver burden interview. Psychiatry and Clinical Neuroscience, 51(5), 281–287. 10.1111/j.1440-1819.1997.tb03199.x 9413874

[cre2415-bib-0003] Barnes, C. M. (2014). Dental hygiene intervention to prevent nosocomial pneumonias. Journal of Evidence Based Dental Practice, 14(Suppl), 103–114. 10.1016/j.jebdp.2014.02.002 24929595

[cre2415-bib-0004] Bekdemir, A. , & Ilhan, N. (2019). Predictors of caregiver burden in caregivers of bedridden patients. Journal of Nursing Research, 27(3), e24. 10.1097/jnr.0000000000000297 PMC6553964 30431539

[cre2415-bib-0005] Gluzman, R. , Meeker, H. , Agarwal, P. , Patel, S. , Gluck, G. , Espinoza, L. , Ornstein, K. , Soriano, T. , & Katz, R. V. (2013). Oral health status and needs of homebound elderly in an urban home‐based primary care service. Special Care in Dentistry, 33(5), 218–226. 10.1111/j.1754-4505.2012.00316.x 23980554

[cre2415-bib-0006] Hestevik, C. H. , Molin, M. , Debesay, J. , Bergland, A. , & Bye, A. (2020). Older patients' and their family caregivers' perceptions of food, meals and nutritional care in the transition between hospital and home care: A qualitative study. BMC Nutrition, 6, 11. 10.1186/s40795-020-00335-w 32206325 PMC7079473

[cre2415-bib-0007] Hirakawa, Y. , Kimata, T. , & Uemura, K. (2013). Current challenges in home nutrition services for frail older adults in Japan – A qualitative research study from the point of view of care managers. Healthcare, 1(1), 53–63. 10.3390/healthcare1010053 27429130 PMC4934505

[cre2415-bib-0008] Hoeksema, A. R. , Peters, L. L. , Raghoebar, G. M. , Meijer, H. J. A. , Vissink, A. , & Visser, A. (2017). Oral health status and need for oral care of care‐dependent indwelling elderly: From admission to death. Clinical Oral Investigations, 21(7), 2189–2196. 10.1007/s00784-016-2011-0 27896484 PMC5559562

[cre2415-bib-0009] Kaplan, V. , Angus, D. C. , Griffin, M. F. , Clermont, G. , Scott Watson, R. , & Linde‐Zwirble, W. T. (2002). Hospitalized community‐acquired pneumonia in the elderly: Age‐ and sex‐related patterns of care and outcome in the United States. American Journal Respiratory Critical Care Medicine, 165(6), 766–772. 10.1164/ajrccm.165.6.2103038 11897642

[cre2415-bib-0010] Khanagar, S. , Naganandini, S. , Tuteja, J. S. , Naik, S. , Satish, G. , & Divya, K. T. (2015). Improving oral hygiene in institutionalised elderly by educating their caretakers in Bangalore City, India: A randomised control trial. Canadian Geriatrics Journal, 18(3), 136–143. 10.5770/cgj.18.145 26495047 PMC4597813

[cre2415-bib-0011] Locher, J. L. , Ritchie, C. S. , Robinson, C. O. , Roth, D. L. , Smith West, D. , & Burgio, K. L. (2008). A multidimensional approach to understanding under‐eating in homebound older adults: The importance of social factors. The Gerontologist, 48(2), 223–234. 10.1093/geront/48.2.223 18483434 PMC2756416

[cre2415-bib-0012] Mahoney, F. I. , & Barthel, D. W. (1965). Functional evaluation: The Barthel index. Maryland State Medical Journal, 14, 61–65. 14258950

[cre2415-bib-0013] Matsusaka, K. , Kawakami, G. , Kamekawa, H. , Momma, H. , Nagatomi, R. , Itoh, J. , & Yamaya, M. (2018). Pneumonia risks in bedridden patients receiving oral care and their screening tool: Malnutrition and urinary tract infection‐induced inflammation. Geriatrics & Gerontology International, 18(5), 714–722. 10.1111/ggi.13236 29380508

[cre2415-bib-0014] Ornstein, K. A. , DeCherrie, L. , Gluzman, R. , Scott, E. S. , Kansal, J. , Shah, T. , Katz, R. , & Soriano, T. A. (2015). Significant unmet oral health needs of homebound elderly adults. Journal of the American Geriatrics Society, 63(1), 151–157. 10.1111/jgs.13181 25537919 PMC4367536

[cre2415-bib-0015] Patterson Norrie, T. , Villarosa, A. R. , Kong, A. C. , Clark, S. , Macdonald, S. , Srinivas, R. , Anlezark, J. , & George, A. (2020). Oral health in residential aged care: Perceptions of nurses and management staff. Nursing Open, 7(2), 536–546. 10.1002/nop2.418 32089850 PMC7024615

[cre2415-bib-0016] Srivastava, G. , Tripathi, R. K. , Tiwari, S. C. , Singh, B. , & Tripathi, S. M. (2016). Caregiver burden and quality of life of key caregivers of patients with dementia. Indian Journal of Psychological Medicine, 38(2), 133–136. 10.4103/0253-7176.178779 27114625 PMC4820552

[cre2415-bib-0017] Tamiya, N. , Noguchi, H. , Nishi, A. , Reich, M. R. , Ikegami, N. , Hashimoto, H. , Shibuya, K. , Kawachi, I. , & Campbell, J. C. (2011). Population ageing and wellbeing: Lessons from Japan's long‐term care insurance policy. Lancet, 378(9797), 1183–1192. 21885099 10.1016/S0140-6736(11)61176-8

[cre2415-bib-0018] Wong, F. M. F. , Ng, Y. T. Y. , & Leung, W. K. (2019). Oral health and its associated factors among older institutionalized residents—A systematic review. International Journal of Environmental Research and Public Health, 16(21), 4132. 10.3390/ijerph16214132 PMC6861909 31717812

[cre2415-bib-0019] Yokobayashi, K. , Matsushima, M. , Watanabe, T. , Fujinuma, Y. , & Tazuma, S. (2014). Prospective cohort study of fever incidence and risk in elderly persons living at home. BMJ Open, 4(7), e004998. 10.1136/bmjopen-2014-004998 PMC4091458 25009132

[cre2415-bib-0020] Yoneyama, T. , Yoshida, M. , Ohrui, T. , Mukaiyama, H. , Okamoto, H. , Hoshiba, K. , Ihara, S. , Yanagisawa, S. , Ariumi, S. , Morita, T. , & Mizuno, Y. (2002). Oral care reduces pneumonia in older patients in nursing homes. Journal of the American Geriatrics Society, 50(3), 430–433. 10.1046/j.1532-5415.2002.50106.x 11943036

[cre2415-bib-0021] Zaitsu, T. , Saito, T. , & Kawaguchi, Y. (2018). The oral healthcare system in Japan. Healthcare, 6(3), 79. 10.3390/healthcare6030079 29996547 PMC6163272

[cre2415-bib-0022] Zarit, S. H. , Reever, K. E. , & Bach‐Peterson, J. (1980). Relatives of the impaired elderly: Correlates of feelings of burden. The Gerontologist, 20(6), 649–655. 10.1093/geront/20.6.649 7203086

[cre2415-bib-0023] Zenthöfer, A. , Meyer‐Kühling, I. , Hufeland, A. L. , Schröder, J. , Cabrera, T. , Baumgart, D. , Rammelsberg, P. , & Hassel, A. J. (2016). Carers' education improves oral health of older people suffering from dementia—Results of an intervention study. Clinical Interventions in Aging, 11, 1755–1762. 10.2147/CIA.S118330 27942206 PMC5137930

