# Supplementary material for: Preliminary investigation of family caregiver burden and oral care provided to homebound older patients
Source: Clin Exp Dent Res. 2021 Mar 8;7(5):840–4. doi: 10.1002/cre2.415 (PMC8543478; doi:10.1002/cre2.415)
Supplement: Supplementary file 1 — Data S1. Contents of the questionnaire. [file CRE2-7-840-s001.docx]

**Appendix.** Contents of the questionnaire

**About family caregiver**

1. Sex □　Male □ Female

2. Age years old

3. Duration of caregiving year(s) month(s)

4. Relationship to the patient □ Spouse □ Child □ Other

4. Interest in oral care for patient □　Yes □ No

5. Caregivers’ concerns during oral care

| 1 | I feel awkward when providing oral care. | Yes No |
| --- | --- | --- |
| 2 | I am too busy to take the appropriate amount of time when providing oral care. | Yes No |
| 3 | The patient feels discomfort during oral care. | Yes No |
| 4 | I do not know how to perform oral care properly. | Yes No |
| 5 | It is difficult for me to remove the patient’s dentures. | Yes No |
| 6 | I feel resistant to working in other people’s mouths. | Yes No |

6. Time spent performing oral care per time minute(s)

7. Care burden: Please circle the response the best describes how you feel.

0: Never 1: Rarely 2: Sometimes 3:Quite frequently 4: Nearly always

| 1 | The patient asks for more help than he/she needs | 0 1 2 3 4 |
| --- | --- | --- |
| 2 | You do not have enough time for yourself | 0 1 2 3 4 |
| 3 | Feel stressed by fulfilling different responsibilities | 0 1 2 3 4 |
| 4 | Are embarrassed by the patient’s behaviour | 0 1 2 3 4 |
| 5 | Feel angry around the patient | 0 1 2 3 4 |
| 6 | Negative effect on other relationships | 0 1 2 3 4 |
| 7 | Feel afraid for the patient’s future | 0 1 2 3 4 |
| 8 | The patient is too dependent | 0 1 2 3 4 |
| 9 | Feel strained around the patient | 0 1 2 3 4 |
| 10 | Your health is affected by caregiving | 0 1 2 3 4 |
| 11 | You have inadequate privacy | 0 1 2 3 4 |
| 12 | Your social life is suffering | 0 1 2 3 4 |
| 13 | Feel uncomfortable having friends | 0 1 2 3 4 |
| 14 | The patient expects you to be the only caregiver | 0 1 2 3 4 |
| 15 | Feel financially stressed | 0 1 2 3 4 |
| 16 | Feel unable to take care of the patient much | 0 1 2 3 4 |
| 17 | Feel a sense of losing control over life | 0 1 2 3 4 |
| 18 | Wish to stop being the patient’s caregiver | 0 1 2 3 4 |
| 19 | Feel uncertain of what to do | 0 1 2 3 4 |
| 20 | Feel you should be doing more for the patient | 0 1 2 3 4 |
| 21 | Feel you could do better for the patient | 0 1 2 3 4 |
| 22 | Feel burdened by providing care to the patient | 0 1 2 3 4 |

**About homebound patient**

1. Age years old

2. What is the homebound patient’s degree of independence in daily life?

Activity Score (Barthel Index)

**FEEDING**

- 0 = unable
- 5 = needs help cutting, spreading butter, etc., or requires a modified diet
- 10 = independent

**BATHING**

- 0 = dependent
- 5 = independent (or uses a shower)

**GROOMING**

- 0 = needs help with personal care
- 5 = independent in tasks related to face/hair/teeth/shaving (implements provided)

**DRESSING**

□ 0 = dependent

□ 5 = needs help, but can do about half unaided

□10 = independent (including buttons, zippers, laces, etc.)

**BOWELS**

□ 0 = incontinent (or needs to be given enemas)

□ 5 = occasional accident

□10 = continent

**BLADDER**

□ 0 = incontinent, or catheterized and unable to manage alone

□ 5 = occasional accident

□ 10 = continent

**TOILET USE**

□ 0 = dependent

□ 5 = needs some help, but can do some things alone

□ 10 = independent (on and off, dressing, wiping) ______

**TRANSFERS (BED TO CHAIR AND BACK)**

□ 0 = unable, no sitting balance

□ 5 = major help (one or two people, physical), can sit

□ 10 = minor help (verbal or physical) 15 = independent ______

**MOBILITY (ON LEVEL SURFACES)**

□ 0 = immobile or < 50 yards

□ 5 = wheelchair independent, including corners, > 50 yards

□ 10 = walks with help of one person (verbal or physical) > 50 yards

□ 15 = independent (but may use any aid; for example, stick) > 50 yards ______

**STAIRS**

□ 0 = unable

□ 5 = needs help (verbal, physical, carrying aid)

□ 10 = independent

**TOTAL (0–100): ___**
